# Two New Rapid Methods of Staining Blood Films

**Published:** 1919

**Authors:** 


### PATHOLOGY AND BACTERIOLOGY

***Two New Rapid Methods of Staining Blood Films.*** By L. TRIBONDEAU. *Comptes Rendus de la Société de Biologie de Paris,* June, 1918.

*First Method*: Two dyes, that the author speaks of as *Dye No. 1* and *Dye No. 2*, are used in this method.

*Dye No. 1* is a neutral solution of eosinate of pure methylene blue.

*Dye No. 2* is an alkaline solution of eosinate of pure methylene blue attacked by ammonia.

*Preparation of Dye No. 1*: Following substances are poured into a glass bottle:

| | |
|---|---|
| Neutral glycerine | 5 cc. |
| Alcohol at 95 per cent. | 45 cc. |
| Pure methylene blue. | 0.20 gr. |
| French eosin | 0.05 gr. |

The bottle is placed in a boiling water bath. Complete mixing is obtained by frequent shaking. As soon as the whole powder is dissolved, the bottle is taken out of the bath, cooled a little, and the preparation is brought to the volume of 50 cc. by addition of alcohol. The whole is filtrated on paper and kept in glass bottles.

*Preparation for Dye No. 2.* 0.20 gr. of pure methylene blue are ground in a mortar with 10 cc. of alcohol at 95 per cent., and mixed in a balloon with 25 cc. glycerine. 4 cc. pure ammonia and 0.05 French eosin, previously ground with alcohol 5 cc., are poured into the balloon. This is heated to 120° C. for 20 minutes, or for 30 minutes in a boiling water bath. After this heating, the volume is brought to 50 cc. by addition of alcohol at 95 per cent. Bottles containing this dye should not be corked until one or two days after preparation.

*Dyeing of the Blood Films.* A thin layer of blood is spread upon a glass slide, and dried by agitation. It is covered with *Dye No. 1*, and this is left to act for 3 minutes. The smear is then washed with distilled water, the excess of water being simply removed by shaking the slide. Two cc. of distilled water are slightly heated in a glass tube, mixed up with 4 drops of *Dye No. 2* and poured warm upon the smear. After 15 minutes, the dye is washed off with water and the drying of the glass slide is rapidly obtained by passing it into a flame, and blowing strongly over it.

A watery tannin solution, at 1 per 20, is then poured on the slide, and immediately washed away, after the color has changed from blue to pink. Drying occurs as formerly.

The smears when properly dyed are mauve or pale pink colored. This method proves extremely convenient to detect all blood parasites.

*Second Method.* The dye used in this method is a *neutral* solution of eosinate of pure methylene blue, attacked by ammonia.

*Preparation.* *a*) 0.20 gr. of pure methylene blue are dissolved in 50 cc. of boiling distilled water. *b*) 0.30 gr. of French eosin are dissolved in 75 cc. of boiling water. *b*) is poured by fractions into *a*). The mixture must be stirred carefully with a glass rod after each pouring of *b*) solution. The drop hanging on to the extremity of the glass rod is deposited after each stirring upon a glass slide. These drops gradually pass from dark blue to pink, and become cloudy. The addition of eosin is stopped as soon as the drop appears pink colored. Then 4 cc. ammonia are added to the preparation, and this is heated to 120° C. After very careful stirring, and complete cooling, the whole is thrown upon a very small paper filter. The precipitate gathered on the filter is dried at 37° C., removed from the paper, ground, and left for 12 hours in contact with absolute alcohol, 90 cc., and glycerine, 10 cc. After another filtration, the dye is placed in glass bottles.

*Dyeing of the Blood Films.* 12 drops of dye are left for 3 minutes on the smear. Again 12 drops of distilled water are mixed up on the slide with the dye by gently moving the slide. After 12 or 15 minutes, the dye is removed, washed off, and the slide dried rapidly by passing it through a flame.

This method has very good results for dyeing distinctly all fine details. But it requires the use of absolutely pure and neutral distilled water.

